# Angiolymphoid hyperplasia with eosinophilia: efficacy of isotretinoin?

**DOI:** 10.1186/1746-160X-2-32

**Published:** 2006-10-04

**Authors:** Fouad El Sayed, Rola Dhaybi, Alfred Ammoury, Myrna Chababi

**Affiliations:** 1 Division of Dermatology, Faculty of Medicine, Lebanese University, Beirut, Lebanon; 2 Department of Dermatology, Purpan University Hospital, Toulouse, France; 3 Department of Pathology, Faculty of Medicine, Lebanese University, Beirut, Lebanon

## Abstract

**Background:**

Angiolymphoid hyperplasia with eosinophilia (ALHE) is a benign but potentially disfiguring vascular lesion. It is usually characterized by dermal and subcutaneous nodules, primarily in the head and neck region. Spontaneous regression is common, but persistent or recurrent lesions may require treatment. Several treatments have been reported but surgery is the most efficient one.

**Methods and results:**

We report a 32-year-old man presenting with multiple nodules on the cheeks, preauricular region and the scalp and who received treatment with isotretinoin (0.5 mg/kg/day) for 1 year with complete resolution of one of his scalp nodules. The rest of the lesions remained stable and were treated with surgical excision without recurrence.

**Conclusion:**

Isotretinoin may play a role in the treatment of ALHE due to its antiangiogenic properties via a reduction of vascular endothelial growth factor (VEGF) production by keratinocytes.

## Background

Angiolymphoid hyperplasia with eosinophilia (ALHE) is a benign but potentially disfiguring vascular lesion. It is usually characterized by dermal and subcutaneous nodules, primarily in the head and neck region. Spontaneous regression is common, but persistent or recurrent lesions may require treatment [1]. Several treatments have been reported but surgery is the most efficient. We report a new case of ALHE and discuss the efficiency of treatment with isotretinoin.

## Methods and results

**Figure 1:**
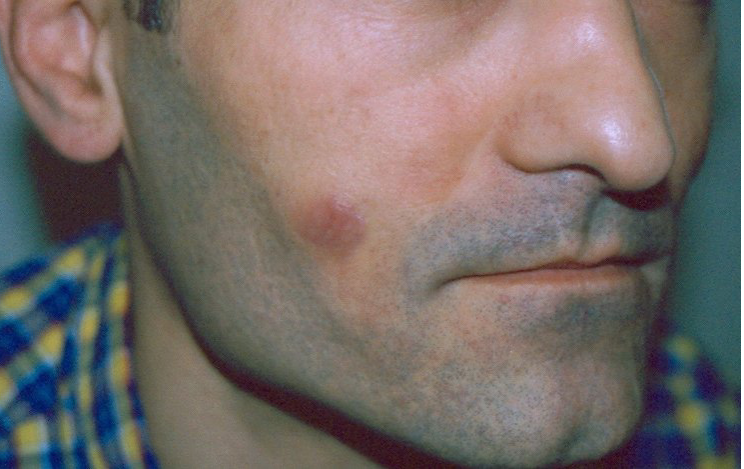
**Photo of one of the nodules**. A 1,5 cm, erythematous nodule on the right cheek.

**Figure 2:**
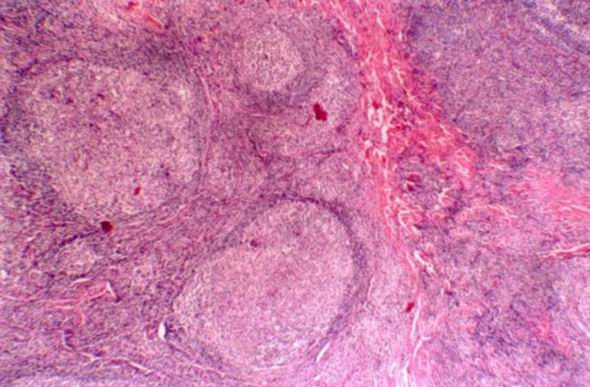
**Histology figure**. Numerous secondary lymphoid follicles (Haematoxylin and eosin, original magnification: × 40).

**Figure 3:**
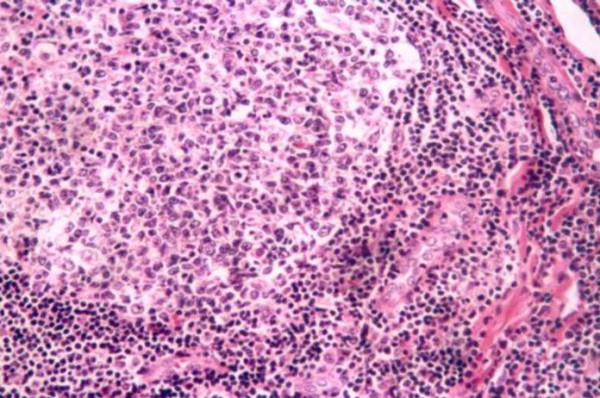
**Histology figure**. Edge of a germinal center showing prominent vascular proliferation with swollen endothelial cells (Haematoxylin and eosin, original magnification: × 200).

**Figure 4:**
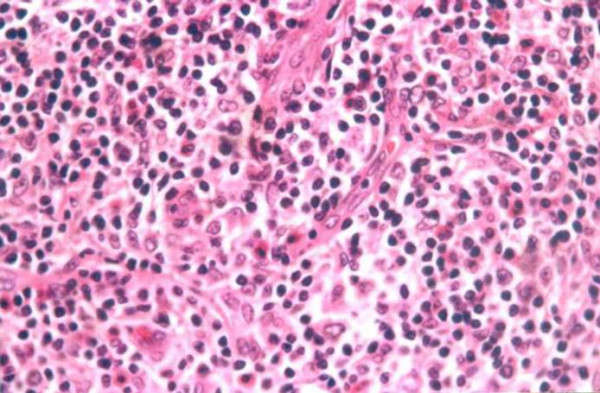
**Histology figure**. Polymorphous inflammatory infiltrate rich in eosinophils (Haematoxylin and eosin, original magnification: × 400).

**Figure 5:**
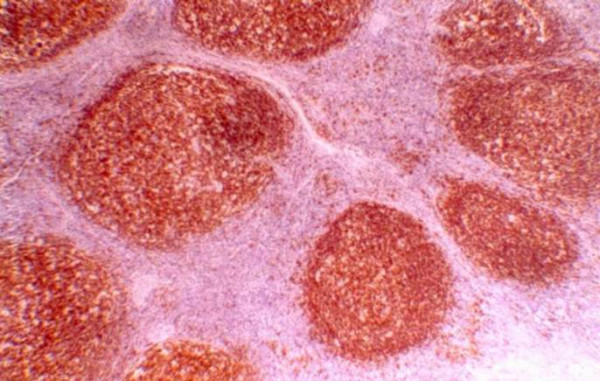
**Immunohistochemistry figure**. CD20 positive lymphoid follicles reflecting the B-Cell nature of the inflammatory cells (Haematoxylin and eosin, original magnification: × 40).

**Figure 6:**
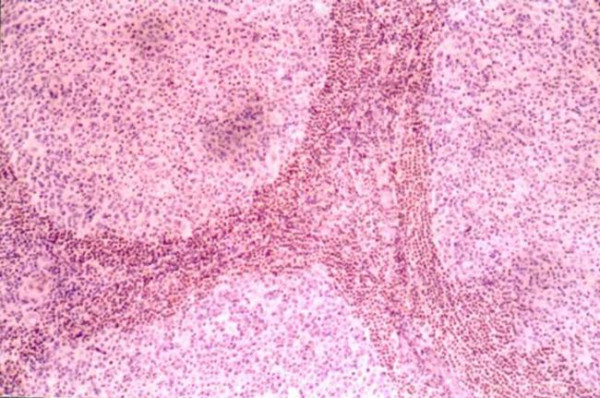
**Immunohistochemistry figure**. Bcl2 negative follicles (Haematoxylin and eosin, original magnification: × 100).

A 32-year-old white man had multiple, pruritic and erythematous nodules on the cheeks, right preauricular region and scalp for 4 years. The lesions increased in number and size throughout these 4 years without spontaneous remission of any of them. The patient was seen by another physician 1 year prior to presentation, was considered to have acne conglobata and received treatment with isotretinoin (0.5 mg/kg/day) for one year. He initially had 2 scalp nodules, 2 nodules on the cheek and 2 in the preauricular region. The disease remained stable during treatment without new lesions. However, there was a complete regression of one of the scalp nodules, the remaining nodules decreased in size but didn't resolve. The improvement was noted within the first 4 months of treatment. Liver function tests and lipid profile remained normal throughout the treatment and he only complained from the common side effects of isotretinoin. At the time of presentation, physical examination revealed 2 erythematous to violaceous brown nodules on the cheeks and 2 other nodules in the right preauricular region and one on the scalp (Fig. 1). There was no regional lymphadenopathy. Routine laboratory investigations including complete blood count, liver function tests, serum IgE level, urinalysis and chest X-ray, were normal. A complete surgical removal of the preauricular nodules was performed. Histopathology of the lesions showed a prominent vascular proliferation involving capillaries, many of which were lined by epithelioid endothelial cells, protruding into the lumen. There was an interstitial and perivascular infiltrate composed primarily of histiocytes and eosinophils with secondary lymphoid follicles (Figs. 2, 3 &4). Immunohistochemical studies showed positive CD20, reflecting the B-cell nature of the infiltrate (Fig. 5) while B-cl2 oncogene was negative (Fig. 6). Based on these findings the diagnosis of ALHE was made. Complete surgical excision of the rest of the nodules was performed and was associated with significant bleeding. The patient continues to remain lesion free 5 years after treatment.

## Discussion

ALHE was first described in 1969 by Wells and Whimster. It is an uncommon benign vascular tumour which presents with small, dull red papules or nodules, 2–3 cm in diameter usually located on the head and neck, mostly in the vicinity of the ear. Other less common involved sites include the trunk, extremities, genitalia, lips and oral mucosa. ALHE affects young to middle-aged adults with a female predominance. Most lesions are solitary although up to 20% are multiple. They are usually asymptomatic but can be pruritic or painful with compression. Some lesions can be pustatile, ulcerated or can bleed spontaneously. Lymphadenopathy occurs in 5% to 20% of patients. Extracutaneous involvement is rare and has been reported in deep soft tissues, salivary glands, orbit, bone, lymph nodes, colon, heart, lungs and generalized involvement has been described. Blood eosinophilia occurs in 10–20% of cases [2-5].

The etiology of ALHE remains unknown. It is either due to a benign neoplastic proliferation of vascular tissue or a reactive hyperplasia of vascular tissue which develops in response to trauma, infections, renin or hyperestrogenic states (pregnancy or oral contraceptives) [3,5]. Eosinophils could be actively involved in the pathogenesis of inflammatory reaction by the production of nitric oxide and eosinophilic cationic protein [6]. Although the inflammatory infiltrate in ALHE appears to be an intrinsic component of the disease, it seems fair to assume that ALHE is a benign neoplastic condition. Kempf et al presented evidence that at least a subset of these lesions may represent a T-cell lymphoproliferative disorder of benign or low-grade malignancy [2,7]. Recently, a malignant transformation has been observed in a young patient with ALHE who developed peripheral T-cell lymphoma [6].

Histopathologically, ALHE involves the dermis and/or the subcutaneous tissue. It shows anomalous proliferation of dilated small to medium-sized blood vessels with a mixed nodular, perivascular, inflammatory infiltrate composed of lymphocytes, eosinophils, histiocytes, plasma cells, neutrophils and mast cells. The lymphocytes are primarily polytypic B lymphocytes. The affected vessels are tubular, elongated or branching and lined by characteristically plump, hobnail endothelial cells which line and protrude into the lumina of blood vessels, resulting in a characteristic "tombstone" appearance. The endothelial proliferation through the vessel walls produces occlusive vascular changes [1,5]. Differential diagnoses of ALHE include cutaneous lymphoma, cavernous hemangioma, pyogenic granuloma, Kaposi's sarcoma, angiomatous lymphoid hamartoma, granuloma faciale, polyarteritis nodosa, pseudolymphoma (lymphocytic infiltrate of Jessner, lymphocytoma cutis), persistent insect bite reaction, injection site granuloma and bacillary angiomatosis [2,5]. In our case, immunohistochemistry rules out other histological differential diagnoses such as epithelioid hemangioendothelioma, B-cell pseudolymphomas, persistent arthropod bite reaction, aluminum-induced granuloma, angiosarcoma and eosinophilic ulcer of the tongue.

ALHE should also be distinguished from Kimura's disease which is a chronic inflammatory condition, producing large subcutaneous nodules on the head and neck with normal overlying skin. Differentiation between these 2 diseases is based on clinical and histology findings (Table 1) [2,3,8].

**Table 1:** Comparison between ALHE and Kimura's disease.

	**ALHE**	**Kimura's disease**
**Presentation**	Superficial papules or nodules Multiple lesions	Large subcutaneous nodules Usually one lesion
**Localisation**	Head and neck	Head and neck
**Population**	Third and fourth decades Caucasian Female	Younger age Asian Male
**Regional lymph nodes**	No	Possible
**Skeletal involvement**	No	Possible
**Blood eosinophilia**	Mild	Marked
**Elevated serum IgE**	Infrequent	Frequent
**Histology**	Marked lymphocytic infiltrate and increased mast cell numbers Exuberant angiomatoid proliferation, masses of uncanalized epithelioid or histiocytoid endothelial cells	Well developed lymphoid follicles Capillary proliferation with large thick-walled vessels
**Origin of the disease**	Vascular origin (endothelial cell)	Chronic inflammatory process

Spontaneous remission is common over the course of months to years, but symptomatic and disfiguring lesions may require treatment [3]. Since the patient noted the resolution of one of the scalp nodules with associated decrease in size of the other lesions within the first months of treatment, the possibility of spontaneous remission becomes less likely. Surgical excision is the treatment of choice, but as the lesions are often multilobulated and poorly delineated, local recurrences occur in 33% to 50% after standard surgical excision. Mohs micrographic surgery with complete margin examination can be considered [2,5]. Surgery can be disfiguring and difficult, especially in the case of periauricular lesions, thus other treatment modalities may be helpful. Treatment options include radiotherapy, curettage, shave excision with electrodessication, cryotherapy, topical, systemic or intralesional corticosteroids, and laser therapy. Continuous-wave carbon dioxide and argon lasers have been successful for treating ALHE but there is a risk of post-treatment scarring. The pulsed dye laser minimizes this risk. Anecdotal reports describe success with intralesional interferon α-2a, indomethacin farnestil, pentoxyfylline, chemotherapeutic agents such as vinblastine, mepolizumab (anti-IL-5) and imiquimod [1-3,9,10].

## Conclusion

Treatment of ALHE with oral retinoids has been mentioned in the medical literature with different results. Marcoux et al reported a 62-year old female with multiple asymptomatic scalp nodules diagnosed as having ALHE, who was treated with acitretin (1 mg/kg/day) and had complete resolution of her scalp nodules after 4 months of treatment [11]. Oh et al reported a 48-year old female with scalp and postauricular nodules who was treated surgically with recurrence of her lesions after 2 months of excision. Thus, a trial of treatment with isotretinoin (30 mg/day) was done with a decrease in size and number of the lesions after 2 months. However, the lesions recurred upon withdrawal of isotretinoin [12]. In our case, isotretinoin was able to stabilize the disease, to eradicate one scalp nodule and to decrease the size of the remaining nodules. This effect may be due to the capacity of retinoids to inhibit angiogenesis via the reduction of vascular endothelial growth factor (VEGF) production by keratinocytes. The anti-activator protein 1 (AP1) activity of the retinoid molecules is responsible for the inhibition of the VEGF expression. In fact, VEGF genes' expression is down-regulated by retinoids via the inhibition of the AP1 pathway [13].

## Competing interests

The author(s) declare that they have no competing interests.
